# A discharge summary adapted to the frail elderly to ensure transfer of relevant information from the hospital to community settings: a model

**DOI:** 10.1186/1471-2318-10-69

**Published:** 2010-09-23

**Authors:** Marie-Jeanne Kergoat, Judith Latour, Isabelle Julien, Marie-Andrée Plante, Paule Lebel, Dominique Mainville, Aline Bolduc, Julie Anne Buckland

**Affiliations:** 1 Institut universitaire de gériatrie de Montréal, Montréal (QC), Canada; 2 Centre hospitalier de l'Université de Montréal, Montréal (QC), Canada

## Abstract

**Background:**

Elderly patients admitted to Geriatric Assessment Units (GAU) typically have complex health problems that require multi-professional care. Considering the scope of human and technological resources solicited during hospitalization, as well as the many risks and discomforts incurred by the patient, it is important to ensure the communication of pertinent information for quality follow-up care in the community setting. Conventional discharge summaries do not adequately incorporate the elements specific to an aging clientele.

**Objective:**

To develop a discharge summary adapted to the frail elderly patient (D-SAFE) in order to communicate relevant information from hospital to community services.

**Methods:**

The items to be included in the D-SAFE have been determined by means of a modified Delphi method through consultation with clinical experts from GAUs (11 physicians and 5 pharmacists) and the community (10 physicians and 5 pharmacists). The consensus analysis and the level of agreement among the experts were reached using a modified version of the RAND^®^/University of California at Los Angeles appropriateness method.

**Results:**

A consensus was reached after two rounds of consultation for all the items evaluated, where none was judged «inappropriate». Among the items proposed, four were judged to be « uncertain » and were eliminated from the final D-SAFE, which was divided into two sections: the medical discharge summary (22 main items) and the discharge prescription (14 main items).

**Conclusions:**

The D-SAFE was developed as a more comprehensive tool specifically designed for GAU inpatients. Additional research to validate its acceptability and practical impact on the continuity of care is needed before it can be recommended for use on a broader scale.

## Background

The province of Quebec has over seven million inhabitants, of whom 14% are aged 65 years or older [1]. The elderly alone mobilize one-half of hospitalization days [2]. The increase in admissions and beds occupied is proportionally more marked in the group aged 75 years and older. Of these older patients, about 15% are admitted to a specialized acute geriatric unit: the Geriatric Assessment Unit (GAU). When admitted to hospital, this clientele of frail elderly often presents an atypical clinical profile, functional decline, and multiple co-morbidities.

Quebec's population enjoys a universal health care insurance plan. Support home care, assessment, and rehabilitation for frail elderly patients are provided by the Health and Social Services Centres of Quebec: an administrative institution combining local community service centres, long-term and rehabilitation care centres, and a general hospital that assumes responsibility for a population in a given territory. The patient has a choice of hospitals. As in the urban centers, the number of hospitals and particularly of specialized university affiliated hospitals is greater; hence, the population is less inclined to visit a single hospital.

Inspired by the British model of acute geriatric unit [3], the Ministry of Health and Social Services implemented this specialized care program in university affiliated hospitals towards the end of the 1970's. This program was originally created to offer, to the frail elderly hospitalized with acute conditions, global and integrated health care in an adapted physical environment, and to ensure a comprehensive assessment and intervention by a multi-professional team [4-6]. Between 1978 and 1999, Geriatric Assessment Units (GAU) were progressively established in all general hospitals. For further information, we refer the reader to a recent publication [7] containing an update on the program activities and available resources, as well as a comparison (similarities and differences) with other varieties of in-hospital geriatric acute or sub-acute programs.

Hospitalization in a GAU represents a turning point in the trajectory of a frail elderly person's health. The inter-professional geriatric evaluation, underlying the comprehensive geriatric assessment [8] and defining clinical GAU care, leads health professionals to elaborate diagnosis and to initiate treatment strategies. The human resources and technology required for such investigation and treatment are often considerable and less accessible in the community setting. Furthermore, given the health risks associated with certain interventions, the possible discomfort to patients, and the necessary mobilization of caregivers, it is undesirable that these interventions be unnecessarily repeated.

In 2004, we conducted a vast study on the quality of GAU clinical processes provided to hospitalized patients after a fall with significant trauma [9]. The content of the discharge summaries that were analysed in that context demonstrated a frequent lack of written information on the following clinical elements: main diagnosis related to the fall (45%), information on cognitive status (49%), balance (81%), lower extremity strength (95%), as well as recommendations for technical aids for walking (60%), homecare (51%), and medical care (22%). Furthermore, the quality of the documents transmitted to the community professionals by the various GAUs differed from one to the other. For example, a detailed discharge summary could be elaborated by the physician, with or without the notes of the other health professionals involved in the patient's hospital care; i.e. the physiotherapist, occupational therapist, dietician, social worker, and liaison nurse. An inter-establishment request form was sometimes added, depending on the region and the follow-up care needed in the community. Considering the amount of time and the expertise invested in the recovery of patients admitted to GAUs during their hospitalization, it seems appropriate to improve the quality of the information transmitted to the family physician and homecare team, so as to ensure that the prescribed treatment strategy is implemented as soon as the patient returns home.

The discharge summary is a communication tool vital to continuity of care, provided the information in the summary is reliable, complete, and received within a reasonable time frame [10-14]. Van Walraven and Rokosh [10] proposed a definition for a quality discharge summary: "*a high-quality discharge summary efficiently communicates information necessary for ongoing care by a patient's family (primary) physician"*. Lack of information can lead to poor continuity of care, resulting in an unnecessary duplication of consultations or investigations, poly-pharmacy, iatrogenic errors, a worsening of the health condition, patient dissatisfaction, and a subsequent loss of confidence in the medical team and physician [15,16]. Furthermore, a dissatisfied patient tends to be less compliant with treatment, with the result that the patient and family seek further consultations with other professionals or choose alternative therapies entirely.

Many studies covering the items and the format of a high-quality discharge summary have already been published [10,12-15,17-30], following a survey of the opinions of the hospital physicians who produced the summaries and of the community physicians who received them. The opinions of the two groups converged as to the content but differed as to the order of priorities. The Collège des médecins du Québec (Quebec Board of Physicians) requires that a discharge summary, containing ten main items [31], be completed for each patient upon departure or, at the latest, 72 hours after the results of the exams essential for the justification of the diagnostic have been added to the file. Community physicians prefer a structured discharge summary of about two pages to a narrative summary [10,17,20,25,27,30]. Certain authors [32] proposed grouping the information concerning the medication on a separate sheet, which could then be used as a discharge prescription. Computerized health care information that includes a summary offers many advantages over hand-written formats: greater readability, easy storage access, and decreased redundancy and transcriptions errors [33,34].

Although the reported studies were conducted in different hospital settings (internal medicine, geriatrics, oncology, with a majority of patients over 65 years old), few studies exist [35] on the need for pertinent information that will ensure effective continuity of care for the frail elderly in their transition from hospital to community-based health services.

The main objective of the project is to propose a Discharge Summary model Adapted for the Frail Elderly patient (D-SAFE) admitted to GAUs, so as to meet the needs of the clinicians who produce and receive these documents. The specific objectives are: 1) to define explicit criteria for the content and format of the discharge summary; 2) to develop, out of these criteria, a discharge summary model to transmit relevant information on the GAU patient to the community physician.

## Methods

**Figure 1:**
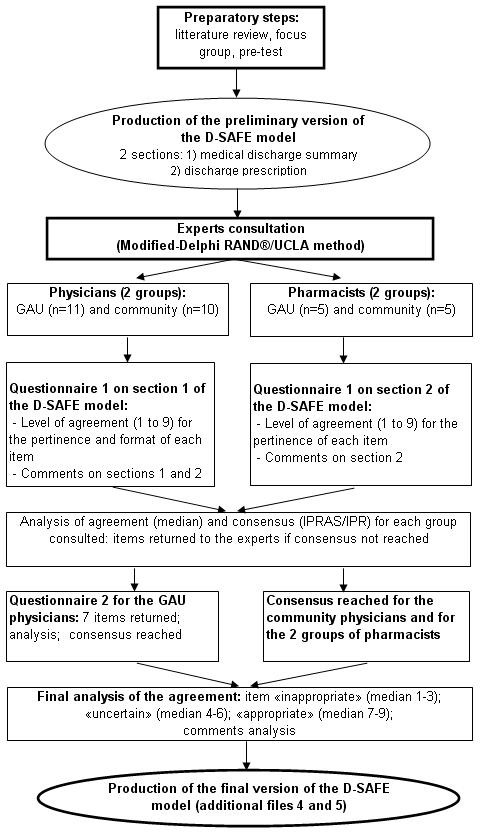
**Steps in the development of the discharge summary model adapted for the frail elderly patient**. Description of the preparatory steps, expert consultation, and production of the final version of the discharge summary model adapted for the frail elderly patient (D-SAFE).

Figure 1 presents the sequence of the project's different stages.

### Preparatory Steps and Production of the Preliminary Version of the D-SAFE

The research team reviewed the literature (up to January 2006) in order to determine the nature and format of the core information to be included in a discharge summary specific to the frail elderly.

In conformity with the general criteria required by the Collège des médecins du Québec, [31], the main information was identified [10,12-15,17-30]: admission diagnosis, main diagnosis (es), other diagnoses and problem(s), complications, treatments, special investigations, living environment after discharge, an update on every active problem, and the patient's condition upon discharge. Also included were the recommended strategies for the patient's problems, prescribed medication (name and dose), identity of the person or establishment receiving the discharge summary, and the dated signature of the treating physician.

Additional pertinent information on the frail elderly was determined mainly from the work of Satzinger et *al *[35], the results of the discharge summary analysis conducted in Quebec GAUs [9], and the research team's clinical experience. This included: social and life-style history (marital status, household vs. nursing-home, etc.); visual, auditory, musculoskeletal, and neurological assessments; functional status [activities of daily living (ADL), instrumental activities of daily living (IADL), continence, mobility and transfer]; nutritional status; cognitive and affective status; psycho-social evaluation; service and care required after discharge; and patient risk of non-compliance to treatment. Certain tools or standardized scales that allow a systematic evaluation of the criteria above, widely used in geriatrics, were also selected: the Mini Mental State Examination (MMSE) [36], the Montreal Cognitive Assessment (MoCA) [37], the Geriatric Depression Scale (GDS) [38], the Timed «Up and Go» [39], the walking speed [40], the Berg score [41], the CASE (Cognitive Assessment Scale for the Elderly) [42], and the SMAF (Functional autonomy measurement system). The latter is a cognitive evaluation tool and a functional autonomy measurement system [43] developed and widely used in the Quebec health care system.

The selected information was then formulated into items. These items were regrouped into two distinct sections: 1) medical discharge summary and 2) discharge prescription. The items were filled out as open-ended questions, multiple choice answers, or qualitative or quantitative results (laboratory data, MMSE, etc.). These items were submitted as a pre-test to four physicians and three pharmacists working in GAUs independently of the research team. Following the pre-test, the sequence and formulation of certain items were modified, and the preliminary version of the discharge summary was produced, comprised of 64 items - 45 in the medical discharge summary and 19 in the discharge prescription.

### Consultation with Experts

A larger group of experts was convened to evaluate the preliminary version of the discharge summary model. It included 11 GAU physicians (4 geriatricians and 7 general practitioners), 10 community general practitioners, as well as five GAU and five community pharmacists. Considering that GAUs are present in most Quebec regions and that 30% of them are located in university affiliated hospitals [9], the GAU physicians and pharmacists were selected separately by means of a stratified random sampling procedure (Table 1) [44]. Specifically, in the case of the GAU physicians, this procedure consisted of first subdividing the GAUs into subgroups according to their geographic region and their university affiliation, each subgroup proportional to its importance in the total distribution. The next step consisted of extracting a random sampling of physicians from each subgroup from the Quebec Geriatric Society list, a professional association of geriatricians and general practitioners, who work mainly with the frail elderly. We then asked each selected GAU physician to recommend a community physician who: 1) had been significantly involved in the care of frail elderly in, at least, the last five years (e.g. a physician of home support service in a health and social services centre), and 2) who had referred patients to GAU.

**Table 1:** Origin of Experts Consulted

	GAU physicians n = 11	Community physicians n = 10	GAU pharmacists n = 5	Community pharmacists n = 5
**Quebec region^1^:**				
Central	6	6	2	3
Periphery	2	2	2	1
Intermediate	2	2	1	1
Remote	1	0	0	0
**University status**				
Yes	5	*n.a*.	2	*n.a*.
No	6	*n.a*.	3	*n.a*.

GAU, Geriatric Assessment Unit; *n.a*. = Not applicable

^1^Central regions = Montreal, Quebec City, and Laval; peripheral regions = Chaudière-Appalaches, Lanaudière, Laurentides, and Montérégie; intermediate regions = Bas Saint-Laurent, Saguenay-Lac-St-Jean, Mauricie-Bois-Franc, Estrie, and Outaouais; remote regions = Abitibi-Témiscamingue and Gaspésie-Îles-de-la-Madeleine.

The GAU pharmacists were first sampled using the list of Quebec GAUs having at least one pharmacist in the multidisciplinary team [9]. Their distribution followed the same process as for the GAU physicians, their ratio being considered in terms of geographical region and university affiliation. The selection of community pharmacists was made upon referral by a GAU pharmacist and was based on their interest in the frail elderly, evidenced by their regular interactions with GAU pharmacists on behalf of their clients.

A RAND^®^-inspired, modified-Delphi type consensus method [45-47] was employed in the expert consultation process. By using a mail-in questionnaire, this method made possible a structured consultation process without the participants having to meet. The anonymity of the participants, with controlled feedback; the statistical evaluation of the group's answers; and the use of additional data constituted the basis for this consensus methodology. The items that did not achieve consensus after the first round were returned to the same group of experts as many times as needed until a consensus was reached. The questionnaires that were sent after the first round were similar to the original but contained additional information, such as the score given by the expert at the first round, the group median, other experts' comments, and a modified "comments section".

The consultation process for this project took place over an eight-month period (July 2007 to March 2008). The physicians were asked to rate their level of agreement on the pertinence and format of each item in the preliminary version of the D-SAFE on a Likert scale ranging from 1 to 9 (1 = total disagreement; 9 = total agreement). The pharmacists completed the same task as to the pertinence of the items in the discharge prescription but were also asked to make qualitative comments on the format of these items. The physicians and pharmacists were also invited to give their impressions of the D-SAFE (pharmacists, only of the discharge prescription) by indicating their general impression; their thoughts on the essential items and those that were unnecessary or missing; as well as their comments on the order and formulation of the items.

### Analysis

The consensus among experts for each item was analyzed and assessed according to two criteria as specified by a modified version of the RAND^®^/University of California at Los Angeles (UCLA) appropriateness method [47]. The first was to evaluate the median value of the rating results of the experts on each item according to three categories: 1) "appropriate", for a value ranging from 7 to 9; 2) "uncertain", for a value ranging from 4 to 6; and 3) "inappropriate", for a value ranging from 1 to 3.

The second was to evaluate the dispersion of the rating results among each expert, using the ratio of the *InterPercentile Range Adjusted for Symmetry *(IPRAS) over the *InterPercentile Range *(IPR), that has shown to be suitable for panels of all sizes. The IPR is «the Interpercentile Range required for disagreement when perfect symmetry exists»; the IPRAS is «the Interpercentile Range Adjusted for Symmetry required for disagreement» [47]. Thus each item requires a different IPRAS, depending on its internal symmetry. Using the RAND^® ^method, the IPR of the rating results was calculated using the 30th and 70th percentile as lower and upper limits, respectively. If the ratio between the IPRAS and the IPR was higher than one, the item attained consensus.

At the end of the first round, the items classified as "uncertain" were modified according to the comments formulated by the experts and were then returned to the panel concerned. The items for which there was no consensus (an IPRAS over IPR ratio inferior to one) were also resubmitted to the experts concerned. For the final analysis, the items selected for the D-SAFE model were those that attained a consensus and that were considered to be appropriate, either by all physicians for the discharge summary section or by all pharmacists for the discharge prescription section.

## Results

### Consensus and Agreement Level

Two rounds were needed to reach a consensus among the four groups of experts; the response rate for both rounds was 100%.

A consensus for each item of the medical discharge summary section of the D-SAFE was reached among the community physicians after the first round (additional files 1 and 2). These physicians judged that all the items were pertinent and were presented in a convenient format. However, after the preliminary round, there were seven items that did not attain a consensus rating among the GAU physicians. These were: as to pertinence, the SMAF (median: 5; IPRAS/IPR: 3.9/6); as to format, life habits (median: 7; IPRAS/IPR: 3.1/5), the SMAF (median: 3; IPRAS/IPR: 2.4/6), the Timed « Up & Go » (median: 5, IPRAS/IPR: 3.1/5), the Berg score (median: 8; IPRAS/IPR: 3.9/6), the presence of chronic pain (median: 5, IPRAS/IPR: 3.1/5), and the GDS (median: 9; IPRAS/IPR: 4.6/5). These seven items were returned to the 11 GAU physicians with additional explanations (the score given by the expert in the first round, the group median, other experts' comments). A consensus was subsequently reached for all items.

In the end, the GAU physicians judged as "uncertain" the pertinence of the SMAF, the walking speed, the Timed "Up & Go", and the GDS (additional file 1). They also judged as "uncertain" the format of the SMAF, the Timed "Up & Go" and chronic pain (additional file 2).

Consensus was obtained for all the items in the prescription section of the D-SAFE. Both the GAU and the community pharmacists considered them to be pertinent after the first round (additional file 3).

### Experts' Comments

Approximately 75% of the physicians consulted (n = 21) considered the D-SAFE to be complete and well adapted to a frail elderly clientele. However, 43% of the respondents considered the document to be lengthy and accordingly believed that this might negatively affect the understanding of the specialized team's work, as well as the global understanding of the patient's condition. In order to counter this perception, the physicians suggested regrouping or moving some items. Approximately 25% of physicians considered some items to be unnecessary; in particular, the results of certain evaluation tools (e.g., the GDS and the walking speed). Other physicians suggested providing a fact sheet, in the event that some community general practitioners were unfamiliar with these measuring tools. Furthermore, approximately 38% of physicians suggested increasing the writing space in three sections: diagnoses, investigations, and the evolution of the problems during hospitalization.

The pharmacists' and physicians' comments on the discharge prescription deemed the prescription to be clear, complete, and pertinent. There was unanimous agreement that the tool was well adapted to a frail elderly clientele. A few modifications were suggested: increasing the space for new or modified medication; adding an item to specify the creatinine clearance value, as well as an item to confirm that the prescription was verified by the pharmacist before discharge.

### Production of the Final Version of the D-SAFE

The four items judged to be of « uncertain » pertinence were eliminated from the medical discharge section. Modifications based on the experts' comments and a new format led to a more concise document. Thus, the medical discharge summary is three pages long, compared to the original five, and the discharge prescription section is one page, front to back. The items included in the final D-SAFE model are presented in Tables 2 (medical discharge summary) and 3 (discharge prescription). This final format for the model is presented in the additional files 4 and 5.

**Table 2:** Final Items in the Medical Discharge Summary Section of the D-SAFE Model

**1. Reason for admission**
**2. Main diagnosis and other active diagnoses **(specify if: allergy, chronic pain, tobacco, alcohol)
**3. Non-active diagnoses**
**4.Social and life-style history upon admission **(marital status, household arrangements, guaranteed income supplement, legal protection measures, services received, etc.)
**5. Pertinent findings of the medical history-taking or the physical exam (specifically, vision, audition, musculoskeletal, and neurological)**
**6. Investigations and consultations **(labs, imaging, other) - *indicate if copies of the reports are annexed to the document*
**7. Mental functions**
7.1. Cognitive status
7.2. Affective status
7.3. Neurobehavioral symptoms associated with dementia
7.4. Facultative: MMSE, MOCA, CASE, Geriatric depression scale (GDS)
**8. Functional status - *indicate if copies of the reports are annexed to the document***
8.1. Activities of daily living
8.2. Instrumental activities of daily living
8.3. Urinary or fecal incontinence
8.4. Mobility/transfer
8.5. Technical support
8.6. Facultative: Walking speed, Timed «Up & Go», Berg score
**9. Nutritional status ***indicate if copies of the reports are annexed to the document*
9.1. Actual weight
9.1. Height
9.3. Weight variation in the past 6 months
9.4. Dysphagia
9.5. Other
**10. Psychosocial assessment ***- indicate if copies of the reports are annexed to the document*
**11. Evolution of clinical problems during hospitalization**
**12. Instructions at discharge and follow-up**
12.1 Medical services (specialists' names, if known)
12.2. Professional care and services
12.2.1. Nurse
12.2.2. Physical therapist
12.2.3. Occupational therapist
12.2.4. Social worker
12.2.5. Dietician
12.2.6. Pharmacist
12.2.7. Respiratory therapist
12.2.8. Foot care
12.3 Programs
12.3.1 Day center
12.3.2 Day hospital
12.3.3 Gerontopsychiatry
12.3.4 Palliative care
12.3.5 Functional and intensive rehabilitation
12.3.6 Other
12.4 Home support services
12.4.1 Household help
12.4.2 Help with meal preparation
12.4.3 Help with errands
12.4.4 Meals on wheels
12.4.5 Accompaniment service
12.4.6 Friendship visits
12.4.7 Orderly support for personal hygiene
12.4.8 Other
12.5 Services for natural caregivers
12.5.1 Respite
12.5.2 Information/counselling service
12.5.3 Psychosocial services
12.5.4 Support groups
12.5.5 Other
12.6 Technical support
12.6.1 Orthotics or prosthetics
12.6.2 Walker
12.6.3 Cane
12.6.4 Wheelchair
12.6.5 Special equipment (bars...)
12.6.6 Incontinence protection
12.6.7 Other
**13. Patient orientation**
12.1. Place of residence
12.2. Relocation (type of structure, name of the establishment, if known)
**14. Additional notes**
**15. Signature of primary hospital physician **(name in print, licence number, date)
**16. Name of family physician**
**17. CLSC attended **(name of establishment, name of case manager)
**18. Resource-person **(name, relationship with the patient, phone number)
**19. Copy given to**
19.1. Patient
19.2. Name of physician or establishment

MMSE, Mini Mental State Examination; MOCA, the Montreal Cognitive Assessment; CASE, Cognitive Assessment Scale for the Elderly; GDS, Geriatric depression scale; CLSC, Centre Local de Services Communautaires/Local community service centre

**Table 3:** Final Items for the Discharge Prescription Section of the D-SAFE Model

**1. Community or institutional pharmacy pre-hospitalization**
1.1. Phone number
1.2. Fax number
**2. Allergies**
**3. Drug intolerances**
**4. CrCl (mL/min)**
4.1. Date
**5. Creatinine**
**6. Weight (Kg)**
6.1. Date
**7. Signature of the pharmacist doing the Rx history**
7.1. Phone number
7.2. Pager
7.3. Date
**8. Medication prior to admission**
8.1. Name
8.2. Comments
8.3. Specify if continuing/modifying/stopping
8.4. Length (number of days)
8.5. Renewal (number)
**9. Changes/new medications at discharge and narcotics**
9.1. Name
9.2. Indications
9.3. Length (number of days)
9.4. Renewal (number)
**10. Specify if weekly pill box**
**11. Barriers to patient's compliance (vision, hearing, manual dexterity, cognition, complex dosing regimen)**
**12. Physician's signature**
12.1. Name in print
12.2. Licence number
12.3. Phone number
12.4. Fax number
12.5. Date
**13. Notes for the community or institutional pharmacist**
**14. Prescription verified by the pharmacist before patient discharge**

CrCl, creatinine clearance; Rx, medication

## Discussion

Many studies have been conducted for the purpose of improving the quality of discharge summaries generally. Given the increasing number of hospitalized elderly patients, many studies have considered a better adapted discharge summary to be necessary in order to improve the quality of information transfer by the hospital to the community, particularly in the case of those patients judged to be the most vulnerable [27,33,48].

Our goal in this project was to produce, in collaboration with community physicians and pharmacists, a discharge summary model in which the items were specifically adapted to the geriatric clientele admitted to GAUs. In the context of our study of the quality of care procedures in GAU, we ascertained significant flaws in the format and content of the transmitted hospital summary [9].

### Strengths and Limitations

The modified-Delphi RAND/UCLA method and the assessment criteria for the items that were chosen for this study are methodological approaches that have already proven effective in the health sector [49-53]. The pertinence of the results obtained through the modified-Delphi method relied mostly on the selected experts. However, it is essential that panel members be sufficient in number (at least 7 to 15 experts) and have a developed expertise in the specific field of study [45,46]. Our 21 physicians and 10 pharmacists fulfilled these requirements.

Most of the items in the D-SAFE's medical discharge summary section attained a high level of agreement among physicians. This can be attributed to the fact that the summary contains the conventional discharge summary data, and that the elements adapted to the geriatric context resulted from a relevant and specific literature review, as well as from the real experience of clinicians in the field. The expertise and practical experience of the GAU professionals, greatly utilized in the outline, as well as for the creation and development of the preliminary D-SAFE model, undoubtedly contributed to the high level of agreement after the first round.

The high level of agreement after the first round on the discharge prescription section can be attributed to the involvement of professionals from the outset and to the urgent clinical need for such a tool that had yet to be met in routine practice. Hence, a tool that is so much more than a simple list of medications for the hospital and community pharmacists was perceived as highly useful and pertinent.

The advantage of this summary discharge lies in its content which covers the main aspects, considered to be essential to geriatric follow-up but heretofore omitted or incomplete in conventional discharge summaries. During the evaluation stage of this project, Halasyamani *et al. *[54] published a discharge checklist for hospitalized elderly patients. Most of the items deemed by this research team to be essential to an elderly patient's discharge summary coincided with those obtained in the D-SAFE: a detailed description of the medical interventions (not simply a medication list), a detailed functional status, as well as recommendations for follow-up. The model that we have developed is different in that it also includes the affective status, nutritional status, social issues, and the patient's needs for technical support and the services provided by natural caregivers. In this way, it allows for a better global understanding of the aging clientele's needs following acute hospitalization that requires specialized care in a GAU. It gives health care professionals the option to add specific items for special cases. Furthermore, the presence of explicit items to be filled out in the D-SAFE raises awareness among hospital physicians as to the importance of the continuity of care after discharge from the GAU.

The format of the D-SAFE was the experts' principal reservation, as they believed it needed improvement. But a more complete discharge summary would necessarily impose a lengthier document. In the end, the incorporation of the comments provided by the experts permitted us to reduce the length of the summary to three pages from the original five. Depending on the complexity of the case, the document could be lengthened. A computerized version of the document, allowing the increase or decrease of the space for each item, would be useful, as it would reflect the complexity of each patient profile and afford its users greater flexibility.

Unfortunately, time and budget constraints limited our research to physicians and pharmacists. Consultation with a wider variety of community health care professionals would have been useful, given that other caregivers, such as the case manager from home care services of a local health board, also receive patient files. These professionals would have brought other important insights and contributions by identifying unaddressed issues that might compromise the continuity of quality care between the hospital and the community care setting.

We are also aware that the timeliness of discharge summary completion and receipt constitutes a major and constant challenge for every hospital. However, our project was not intended to address this issue but rather to focus on developing the content and format of the discharge summary. Other steps regarding the various aspects of this tool - production, usefulness, acceptance, cost, etc. - will follow.

### D-SAFE Utility

In Quebec hospitals, the discharge summary is dictated by inpatients' physicians and is sent from the medical records department by mail or fax. In about half the GAU, the liaison nurse on staff faxes the discharge summary before a patient's departure. We hope that this new summary discharge model which combines dictation and multiplied check options will actually save GAU physicians time. Although we suspect (without any monitoring) that a discharge summary in GAU is generally lengthier than that of any other medical or surgical services, experience in geriatric medicine has taught us that taking time now could save time in the long run.

As professionals who have been involved in the field of geriatric medicine for many years, we observe that, despite the dramatic increase in the number and percentage of frail elderly patients in our society (and proportionally even more in hospitals), health organisations and health professionals in hospitals and the community are reluctant to put in place adapted measures that will respond to the specific needs of this clientele. In our opinion, the D-SAFE can be recommended for use in general medicine hospital units when two prerequisites are met: 1) there must be a multidimensional approach to care, carried out by an inter-professional team; and 2) the hospital stay must be long enough (usually more than seven days) in order to collect the relevant information. The D-SAFE will be particularly relevant when used in hospitals not having an acute geriatric unit, but serving a clientele of frail elderly patients if they meet the above two criteria.

The D-SAFE will also interest the general practitioner in the community who might not have a specific interest in this clientele: its added usefulness as a promotional and educational tool will underline the important dimension of well-being and health that might not automatically be considered. Finally, we strongly recommend the use of the prescription summary section to all physicians and pharmacists taking care of frail elderly, irrelevant of setting.

### Conclusion and Perspectives

We believe our discharge summary model to be more informative in terms of the particular health issues faced by the frail elderly admitted to GAU, in comparison to the conventional document used for patients generally. Before implementing the D-SAFE in other geriatric settings or hospital services, we recommend that each milieu use it first as a prototype and then modify it according to its specific requirements. The final version of the D-SAFE model will be assessed in an open study that will address user satisfaction.

## Competing interests

The authors declare that they have no competing interests.

## Authors' contributions

MJK directed and supervised all steps of the study. MJK, JL, AB, JAB and PL drafted the manuscript. MAP wrote the first draft of the discharge prescription section of the D-SAFE. DM and AB analyzed the data. All the authors participated in the design and execution of the study. All the authors reviewed and approved the manuscript.

## Pre-publication history

The pre-publication history for this paper can be accessed here:

http://www.biomedcentral.com/1471-2318/10/69/prepub

## Supplementary Material

Additional file 1 **Level of agreement on the pertinence of the items in the medical discharge summary section of the final D-SAFE model**. Results for the pertinence of the items in the medical discharge summary section of the D-SAFE model. Click here for file

Additional file 2 **Level of agreement on the format of the items in the medical discharge summary section of the final D-SAFE model**. Results for the format of the items in the medical discharge summary section of the D-SAFE model. Click here for file

Additional file 3 **Level of agreement on the pertinence of the items in the discharge prescription section of the final D-SAFE model**. Results for the pertinence of the items in the discharge prescription section of the D-SAFE model. Click here for file

Additional file 4 **Discharge summary model adapted to the frail elderly patient - Medical discharge summary**. Final version of section 1 of the D-SAFE model. Click here for file

Additional file 5 **Discharge summary model adapted to the frail elderly patient - Discharge prescription**. Final version of section 2 of the D-SAFE model. Click here for file
