# Supplementary material for: A discharge summary adapted to the frail elderly to ensure transfer of relevant information from the hospital to community settings: a model
Source: BMC Geriatr. 2010 Sep 23;10:69. doi: 10.1186/1471-2318-10-69 (PMC2955597; doi:10.1186/1471-2318-10-69)
Supplement: Additional file 1 — Level of agreement on the pertinence of the items in the medical discharge summary section of the final D-SAFE model. Results for the pertinence of the items in the medical discharge summary section of the D-SAFE model. [file 1471-2318-10-69-S1.PDF]

Additional file 1. Level of agreement on the pertinence of the items in the medical discharge summary section of the final D-SAFE model

Items	Physicians Total (n=21)		GAU physicians (n=11)	Community physicians (n=10)
	Median of the level of agreement (IPRAS/IPR)			
Resource-person	9 (7.6/1)		8 (6.9/2)	9 (8.4/0)
CLSC of belonging	8 (7.6/1)		8 (6.9/2)	9 (7.6/1)
Case manager	8 (6.9/2)		8 (6.9/2)	9 (6.9/2)
Reason for admission	9 (8.4/0)		9 (8.4/0)	9 (8.4/0)
Main diagnosis	9 (8.4/0)		9 (8.4/0)	9 (8.4/0)
Other diagnosis(es)	9 (8.4/0)		9 (8.4/0)	9 (8.4/0)
Problems and/or complications	9 (8.4/0)		9 (8.4/0)	9 (8.4/0)
Life-style habits (Tobacco, alcohol, etc.)	7 (5.4/4)		7 (3.9/2)	9 (7.4/1.3)
Allergies	9 (8.4/0)		9 (8.4/0)	9 (8.4/0)
Social history	9 (6.9/2)		7 (6.9/2)	9 (8.1/0.3)
Pertinent findings based on the medical history or the physical exam	9 (7.6/1)		9 (7.6/1)	9 (8.4/0)
Functional status	9 (8.4/0)		9 (8.4/0)	9 (8.4/0)
ADLs	9 (8.4/0)		9 (7.6/1)	9 (8.4/0)
IADLs	9 (8.4/0)		9 (7.6/1)	9 (8.4/0)
Urinary and/or fecal incontinence	9 (8.4/0)		9 (7.6/1)	9 (8.4/0)
SMAF	7 (5.4/4)		5 (3.1/3)	8.5 (6.6/2.3)
Mobility/transfer assessment	9 (7.6/1)		8 (6.9/2)	9 (8.1/0.3)
Mobility	8 (7.6/1)		8 (6.1/1)	9 (8.1/0.3)
Transfer	9 (7.6/1)		8 (6.9/2)	9 (8.1/0.3)
Walking speed	7 (4.6/3)		6 (3.9/2)	8 (5.9/3.3)
TUG	6 (3.9/2)		5 (3.1/3)	6.5 (5.4/2.6)
Berg	7 (4.6/3)		7 (3.1/3)	8 (5.9/3.3)

Chronic pain	9 (6.9/2)		7 (5.4/4)	9 (8.4/0)
Nutritional status	9 (7.6/1)		8 (6.9/2)	9 (8.1/0.3)
Actual weight	9 (7.6/1)		8 (7.6/1)	9 (8.1/0.3)
Height	8 (6.9/2)		8 (6.9/2)	8.5 (6.9/2)
Weight variation in the past 6 months	8 (6.9/2)		8 (6.1/1)	9 (7.4/1.3)
Dysphagia	9 (6.9/2)		7 (6.1/3)	9 (8.1/0.3)
Mental functions	9 (7.6/1)		9 (7.6/1)	9 (8.4/0)
MMSE	9 (7.6/1)		9 (7.6/1)	9 (8.4/0)
MOCA	8 (6.9/2)		8 (6.1/1)	9 (7.6/1)
PECPA-2R	8 (6.9/2)		8 (6.9/2)	7.5 (6.4/2.6)
Neurobehavioral symptoms associated to dementia	9 (7.6/1)		9 (6.9/2)	9 (8.1/0.3)
GDS	6 (4.6/3)		6 (3.9/2)	7 (5.1/2.3)
Investigations (labs, imaging, other)	9 (8.4/0)		9 (7.6/1)	9 (8.4/0)
Consultations	9 (7.6/1)		9 (6.9/2)	9 (8.4/0)
Problem evolution in the hospital	9 (8.4/0)		9 (6.9/2)	9 (8.4/0)
Recommendations and follow-up	9 (8.4/0)		9 (8.4/0)	9 (8.4/0)
Medical services	9 (8.4/0)		9 (8.4/0)	9 (8.4/0)
Professional care and services	9 (7.6/1)		9 (8.4/0)	9 (7.6/1)
Home support services	9 (8.4/0)		9 (8.4/0)	9 (8.1/0.3)
Services for natural caregivers	9 (7.6/1)		9 (7.6/1)	9 (7.4/1.3)
Technical support	9 (8.4/0)		9 (7.6/1)	9 (8.4/0)
Programs	9 (7.6/1)		9 (7.6/1)	8.5 (7.4/1.3)
Patient orientation	9 (8.4/0)		9 (7.6/1)	9 (8.4/0)

CLSC, Centre Local de Services Communautaires/Local community service centre; ADLs, Physical Activities of Daily Living; IADLs, Instrumental Activities of Daily Living; SMAF, functional autonomy measurement system; TUG, Timed « Up & Go »; Berg, Berg scale; MMSE, Mini-Mental state examination; MOCA, The Montreal cognitive assessment; PECPA-2R, Cognitive assessment of the elderly protocol; GDS, Geriatric depression scale
