# Supplementary material for: A discharge summary adapted to the frail elderly to ensure transfer of relevant information from the hospital to community settings: a model
Source: BMC Geriatr. 2010 Sep 23;10:69. doi: 10.1186/1471-2318-10-69 (PMC2955597; doi:10.1186/1471-2318-10-69)
Supplement: Additional file 2 — Level of agreement on the format of the items in the medical discharge summary section of the final D-SAFE model. Results for the format of the items in the medical discharge summary section of the D-SAFE model. [file 1471-2318-10-69-S2.PDF]

Additional file 2. Level of agreement on the format of the items in the medical discharge summary section of the final D-SAFE model

Items	Physician Total (n=21)		GAU physician (n=11)	Community physician (n=10)
	Median of the level of agreement (IPRAS/IPR)			
Resource-person	*n.a*		*n.a*	*n.a*
CLSC of belonging	*n.a*		*n.a*	*n.a*
Case manager	*n.a*		*n.a*	*n.a*
Reason for admission	*n.a*		*n.a*	*n.a*
Main diagnosis	*n.a*		*n.a*	*n.a*
Non-active diagnosis(es)	*n.a*		*n.a*	*n.a*
Problems and/or complications	*n.a*		*n.a*	*n.a*
Life-style habits (tobacco, alcohol, etc.)	7 (5.4/2)		7 (5.4/2)	7.5 (5.7/3.6)
Allergies	9 (7.6/1)		8 (6.9/2)	9 (8.4/0)
Social history	9 (6.1/3)		8 (6.9/2)	9 (5.9/3.3)
Pertinent findings during the medical history taking or the physical exam	9 (7.6/1)		8 (7.6/1)	9 (7.6/1)
Functional status	9 (7.6/1)		8 (6.9/2)	9 (8.1/0.3)
ADLs	9 (6.9/2)		8 (6.9/2)	9 (8.1/0.3)
IADLs	9 (6.9/2)		8 (6.9/2)	9 (8.1/0.3)
Urinary and/or fecal incontinence	9 (8.4/0)		9 (7.6/1)	9 (8.4/0)
SMAF	5 (3.9/2)		5 (3.1/1)	7 (5.4/2.6)
Mobility/transfer assessment				
Mobility	9 (7.6/1)		9 (7.6/1)	9 (8.1/0.3)
Transfer	9 (7.6/1)		8 (6.9/2)	9 (8.1/0.3)
Walking speed	8 (5.4/4)		8 (5.4/4)	8 (5.9/2)
TUG	7 (5.4/2)		6 (3.9/2)	7.5 (6.1/1.6)
Berg	7 (5.4/2)		7 (6.1/1)	7 (5.4/2.6)
Chronic pain	8 (6.1/3)		6 (4.6/3)	9 (7.4/1.3)
Nutritional status				
Actual weight	*n.a*		*n.a*	*n.a*

Height	*n.a*		*n.a*	*n.a*
Weight variation in the past 6 months	9 (7.6/1)		8 (6.1/3)	9 (8.1/0.3)
Dysphagia	8 (7.6/1)		8 (6.1/3)	9 (7.6/1)
Mental functions				
MMSE	9 (8.4/0)		9 (8.4/0)	9 (8.4/0)
MOCA	9 (7.6/1)		9 (7.6/1)	9 (7.4/1.3)
PECPA-2R	9 (7.6/1)		9 (7.6/1)	9 (7.4/1.3)
Neurobehavioral symptoms associated to dementia	9 (7.6/1)		9 (7.6/1)	9 (8.4/0)
GDS	9 (6.1/3)		9 (6.1/3)	8.5 (7.2/1.6)
Investigations (labs, imaging, other)	9 (7.6/1)		8 (7.6/1)	9 (8.4/0)
Consultations	9 (7.6/1)		9 (7.6/1)	9 (8.1/0.3)
Problem evolution in the hospital	9 (7.6/1)		8 (6.9/2)	9 (8.1/0.3)
Recommendations and follow-up				
Medical services	9 (8.4/0)		9 (7.6/1)	9 (8.4/0)
Professional care and services	9 (8.4/0)		9 (7.6/1)	9 (8.4/0)
Home support services	9 (8.4/0)		9 (6.9/2)	9 (8.4/0)
Services for natural caregivers	9 (8.4/0)		9 (6.9/2)	9 (8.4/0)
Technical support	9 (8.4/0)		9 (6.9/2)	9 (8.4/0)
Programs	9 (8.4/0)		9 (6.9/2)	9 (8.4/0)
Patient orientation	9 (8.4/0)		9 (6.9/2)	9 (8.4/0)

n.a, not applicable; CLSC, Centre local de Services Communautaires/Local community service centre; ADLs, Physical Activities of Daily Living; IADLs, Instrumental Activities of Daily Living; SMAF, Functional autonomy measurement system; TUG, Timed « Up & Go »; Berg, Berg scale; MMSE, Mini-Mental state examination; MOCA, The Montreal cognitive assessment; PECPA-2R, Cognitive assessment of the elderly protocol; GDS, Geriatric depression scale
