# Supplementary material for: A discharge summary adapted to the frail elderly to ensure transfer of relevant information from the hospital to community settings: a model
Source: BMC Geriatr. 2010 Sep 23;10:69. doi: 10.1186/1471-2318-10-69 (PMC2955597; doi:10.1186/1471-2318-10-69)
Supplement: Additional file 3 — Level of agreement on the pertinence of the items in the discharge prescription section of the final D-SAFE model. Results for the pertinence of the items in the discharge prescription section of the D-SAFE model. [file 1471-2318-10-69-S3.PDF]

Additional file 3. Level of agreement on the pertinence of the items in the discharge prescription section of the final D-SAFE model

Items	Pharmacists Total (n=10)		GAU pharmacists (n=5)	Community pharmacists (n=5)
	Median of the level of agreement (IPRAS/IPR)			
Community or institution pharmacy pre-hospitalization	9 (7.6/1)		9 (8.4/0)	8 (7.5/0.8)
Phone number	9 (7.6/1)		9 (7.8/0.8)	9 (7.8/0.8)
Fax number	8 (6.1/1.6)		8 (5.7/1.6)	8 (6.9/1.6)
Weight (Kg)	9 (7.4/1.3)		9 (7.8/0.8)	9 (7.2/1.6)
Allergies	9 (8.4/0)		9 (8.4/0)	9 (8.4/0)
CrCl (mL/min)	9 (8.4/0)		9 (8.4/0)	9 (8.4/0.01)
Date	9 (8.4/0)		9 (8.4/0)	9 (8.4/0)
Drug intolerances	9 (8.4/0)		9 (8.4/0)	9 (8.4/0)
Section A	9 (8.4/0)		9 (8.4/0)	9 (8.4/0)
Medication prior admission	9 (8.4/0)		9 (8.4/0)	9 (8.4/0)
Comments	9 (7.4/1.3)		9 (8.4/0)	7 (6.6/1.6)
Action taken : Continue/Modify/Stop	9 (8.4/0)		9 (8.4/0)	9 (8.4/0)
Length (number of days)	9 (8.4/0)		9 (7.8/0.8)	9 (8.4/0)
Renewal (number)	9 (8.4/0)		9 (8.4/0.01)	9 (8.4/0)
Section B	9 (8.4/0)		9 (8.4/0)	9 (8.4/0)
Changes/new medications at discharge and narcotics	9 (8.4/0)		9 (8.4/0)	9 (8.4/0)
Indications	9 (8.4/0)		9 (8.4/0)	9 (8.4/0)
Length (number of days)	9 (8.4/0)		9 (8.4/0)	9 (8.4/0)
Renewal (number)	9 (8.4/0)		9 (8.4/0)	9 (8.4/0)
Section C- Weekly pill box	9 (8.4/0)		9 (8.4/0)	9 (8.4/0)
Section D- Notes to the community or institution pharmacist	9 (7.6/1)		9 (7.8/0.8)	9 (7.8/0.8)
Barriers to patient's compliance (vision, hearing, manual dexterity, cognition, complex dosing regimen)	8.5 (7.6/1)		8 (7.5/0.8)	9 (7.8/0.8)
