# Supplementary material for: A discharge summary adapted to the frail elderly to ensure transfer of relevant information from the hospital to community settings: a model
Source: BMC Geriatr. 2010 Sep 23;10:69. doi: 10.1186/1471-2318-10-69 (PMC2955597; doi:10.1186/1471-2318-10-69)
Supplement: Additional file 4 — Discharge summary model adapted to the frail elderly patient - Medical discharge summary. Final version of section 1 of the D-SAFE model. [file 1471-2318-10-69-S4.PDF]

Discharge summary model adapted for the frail elderly patient

Medical discharge summary

iugm
Institut universitaire de gériatrie de Montréal

Confidential

« Addressograph »

Problem(s) justifying the admission

Main diagnosis and other active diagnoses (specify if : allergies, chronic pain, tobacco, alcohol)

Non-active diagnoses

Social and life-style history upon admission (marital status, household arrangements, level of income, legal protection measures, services received, etc.)

Pertinent findings based on the medical history taking or physical exam (specifying : vision, audition, musculoskeletal and neurological systems)

Research centre, IUGM, copyright © 2009

Investigations (labs, imaging, other) and consultations ❑ Joint copy(ies) of the report(s) to the document

Mental functions ❑ Normal ❑ Joint copy(ies) of the report(s) to the document

Cognitive status :

Affective status :

❑ Neurobehavioral symptoms associated with dementia

Other :

Functional status ❑ Joint copy(ies) of the report(s) to the document

Incontinence ❑ urinary ❑ fecal

IADLs :

ADLs :

Mobility/transfer :

Technical support:

Nutritional status ❑ Joint copy(ies) of the report(s) to the document

Actuel weight : Height :

❑ Weight variation in the past 6 months ❑ Dysphagia

Other :

Psychosocial assessment ❑ Not relevant

Patient's medical complaints addressed during hospitalization, problems, complications and treatments

Research centre, IUGM, copyright © 2009

Instructions at discharge and follow-up

Medical services (specialist's name if known) and f/u appointments:	Professional care and services
	❑ Nurse<br>❑ Dietician<br>❑ Occupational therapist<br>❑ Respiratory therapist<br>❑ Social worker<br>❑ Physiotherapist<br>❑ Pharmacist (medication supervision)<br>❑ Foot care<br>❑ Other:
**Programs**	**Home care services**
❑ Day center<br>❑ Gerontopsychiatry<br>❑ Functional and intensive rehabilitation<br>❑ Day hospital<br>❑ Palliative care<br>❑ Other :	❑ House-keeping<br>❑ Meals on wheels<br>❑ Friendship visits<br>❑ Accompaniment service<br>❑ Meal preparation<br>❑ Personal hygiene<br>❑ Other :
**Services for informal caregivers**	**Technical support**
❑ Respite<br>❑ Information/counseling service<br>❑ Psychosocial services<br>❑ Support groups<br>❑ Other :	❑ Orthotics or prosthetics<br>❑ Walker<br>❑ Cane<br>❑ Incontinence protection<br>❑ Special equipment<br>❑ Wheelchair<br>❑ Other :

Patient orientation

❑ Place of residence or ❑ Relocation

Type of structure :

Name of the establishment (if known) :

Additional notes (studies to pursue for incidental problems found during admission, pending data not yet back at discharge, code status, preferred intensity of care, etc.)

Primary hospital physician signature

Name in print:

Signature :

Licence no. :

Date :

Family physician

Name :

CLSC of belonging

Name of the establishment:

Case manager (name and phone number):

Provider of major social support

Name and relationship to the patient :

Phone number :

Copy given to :

- ❑ Patient
- ❑ Physician name or establishment :

❑ Discharge prescription is joined

Research centre, IUGM, copyright © 2009
