# Supplementary material for: A discharge summary adapted to the frail elderly to ensure transfer of relevant information from the hospital to community settings: a model
Source: BMC Geriatr. 2010 Sep 23;10:69. doi: 10.1186/1471-2318-10-69 (PMC2955597; doi:10.1186/1471-2318-10-69)
Supplement: Additional file 5 — Discharge summary model adapted to the frail elderly patient - Discharge prescription. Final version of section 2 of the D-SAFE model. [file 1471-2318-10-69-S5.PDF]

Discharge summary model adapted for the frail elderly patient

Discharge prescription

« Addressograph »

Community or institution pharmacy pre-hospitalization : Phone number : ( ) Fax number : ( )

Allergies : Drug intolerances :

CrCl (mL/min) : /Date : Creatinine (mmol/L): /Date : Weight (Kg) : /Date :

Signature of pharmacist doing the Rx history; Phone number : Pager number : Date :

A	MEDICATION PRIOR ADMISSION	COMMENTS	ACTION REQUIRED	LENGTH (# days)	RENEWAL (number)
1			Continue Modify Stop		
2			Continue Modify Stop		
3			Continue Modify Stop		
4			Continue Modify Stop		
5			Continue Modify Stop		
6			Continue Modify Stop		
7			Continue Modify Stop		
8			Continue Modify Stop		
9			Continue Modify Stop		
10			Continue Modify Stop		
11			Continue Modify Stop		
12			Continue Modify Stop		

B	CHANGES/NEW MEDICATIONS AT DISCHARGE AND NARCOTICS	INDICATIONS	LENGTH (# days)	RENEWAL (number)
1				
2				
3				
4				
5				
6				
7				

C **WEEKLY PILL BOX** ☐

BARRIERS TO PATIENT'S COMPLIANCE: ☐ Vision ☐ Hearing ☐ Manual dexterity ☐ Cognition ☐ Complex dosing regimen

Physician's signature :	Licence number:	Date :
Name (in print) :	Phone number:	Fax number :

D	NOTES FOR THE COMMUNITY OR INSTITUTION PHARMACIST

❑ Prescription verified by the pharmacist before patient discharge

Research centre, IUGM, copyright © 2009 Confidential

Notes:

1. **The treating physician must complete** SECTION A
   (Rx continued, modified or stopped by encircling each item)

   + SECTION B

   + specify **the duration of treatment and number of renewals.**

2. Narcotics **can not be renewed.**
   Specify the total quantity to dispense
   (ex.: 100 pills, dispense 25 once a week)

Research centre, IUGM, copyright © 2009
Confidential
Adapted from : Ordonnance pharmaceutique de départ du CUSM (MRC0000 02-2002)

2010-09-23 File no. :
